# Effect of Different Compatibilizers on Sustainable Composites Based on a PHBV/PBAT Matrix Filled with Coffee Silverskin

**DOI:** 10.3390/polym10111256

**Published:** 2018-11-12

**Authors:** Fabrizio Sarasini, Francesca Luzi, Franco Dominici, Gianluca Maffei, Annalaura Iannone, Antonio Zuorro, Roberto Lavecchia, Luigi Torre, Alfredo Carbonell-Verdu, Rafael Balart, Debora Puglia

**Affiliations:** 1 Department of Chemical Engineering Materials Environment, Sapienza-Università di Roma, 00184 Rome, Italy; fabrizio.sarasini@uniroma1.it (F.S.); gianluca.maffei@uniroma1.it (G.M.); annalaura.iannone@uniroma1.it (A.I.); antonio.zuorro@uniroma1.it (A.Z.); roberto.lavecchia@uniroma1.it (R.L.); 2 Civil and Environmental Engineering Department, University of Perugia, UdR INSTM, Strada di Pentima 4, 05100 Terni, Italy; francesca.luzi85@gmail.com (F.L.); francodominici1@gmail.com (F.D.); luigi.torre@unipg.it (L.T.); 3 Technological Institute of Materials (ITM), Universitat Politècnica de València (UPV), Plaza Ferrándiz y Carbonell 1, 03801 Alicante, Spain; alcarve1@epsa.upv.es (A.C.-V.); rbalart@mcm.upv.es (R.B.)

**Keywords:** poly(butyleneadipate-*co*-terephthalate, poly(3-hydroxybutyrate-*co*-3-hydroxyvalerate), recycling, coffee silverskin, sustainable development, plasticizers, silane, biodegradable composites

## Abstract

This work investigates the feasibility of using coffee silverskin (CSS), one of the most abundant coffee waste products, as a reinforcing agent in biopolymer-based composites. The effect of using two compatibilizers, a maleinized linseed oil (MLO) and a traditional silane (APTES, (3-aminopropyl)triethoxysilane), on mechanical and thermal behavior of sustainable composites based on a poly(butylene adipate-co-terephthalate/Poly(3-hydroxybutyrate-co-3-hydroxyvalerate) PBAT/PHBV blend filled with coffee silverskin, in both the as-received state and after the extraction of antioxidants, was studied. Thermal (by differential scanning calorimetry), mechanical (by tensile testing), and morphological properties (by scanning electron microscopy) of injection molded biocomposites at three different weight contents (10, 20, and 30 wt %) were considered and discussed as a function of compatibilizer type. The effects of extraction procedure and silane treatment on surface properties of CSS were investigated by infrared spectroscopy. Obtained results confirmed that extracted CSS and silane-treated CSS provided the best combination of resistance properties and ductility, while MLO provided a limited compatibilization effect with CSS, due to the reduced amount of hydroxyl groups on CSS after extraction, suggesting that the effects of silane modification were more significant than the introduction of plasticizing agent.

## 1. Introduction

Drivers such as sustainability, energy efficiency, reduced waste generation, and greenhouse gas emission are emerging powerfully in the current industrial economy, though it is still dominated by a linear and extract-process-consumption disposal philosophy that makes it highly and inherently unsustainable [1]. On the contrary, the recently introduced circular economy model aims at sustainable development by reducing waste and maintaining the value of resources and products for as long as possible, extracting the maximum value from them whilst in use, then recovering and regenerating products and materials at the end of their service life. This goal can be accomplished by using renewable energy, by limiting toxic chemicals, by developing bio-benign products, and finally by elimination of waste [2].

In this context, bio-based sourcing of plastics along with waste valorization approaches could significantly contribute to the adoption of a circular economy model. As regards the waste valorization to high-value added products, agro-food waste presents profitable opportunities due to its great world-wide availability. Therefore, it is of utmost importance the possibility to develop highly sustainable composite materials that combine biopolymers as matrix and agro-industrial residues as fillers. A major source of residues is represented by the coffee industry in the form of defective beans, coffee silverskin (CSS), and spent coffee grounds (SCG), which indeed can cause severe contamination and environmental problems if discarded in landfills [3,4]. It is not surprising that over the last years several efforts have been made to valorize the by-products resulting from coffee processing. SCG have been extensively investigated as filler in composite materials [5,6,7,8,9,10], as source of important ingredients such as oil, terpenes, caffeine, and polyphenols [11,12], or more recently as a source for Quantum Dots [13]. Indeed, comparatively less attention has been paid to the major by-product of the coffee roasting process, i.e. coffee silverskin, which is a thin tegument forming the outer layer of coffee beans that is discarded due to the expansion of the beans occurring during roasting [4,14]. Traditional uses of CSS include mainly compost and soil fertilization but, more recently, its chemical composition rich in dietary fibers, phenolic compounds, and melanoidins, aroused a lot of interest in food, cosmetic and pharmaceutical industries [15]. Unfortunately, these methods cannot be considered the most efficient in terms of value addition, especially in view of the large availability of such residues, which is estimated at around 2 billion tons per year [16].

In this framework, a sustainable exploitation of these residues is their use as filler in new biodegradable materials, which has been rarely addressed in literature [10,17]. In a previous work [17], the feasibility of using coffee silverskin as a reinforcing agent in biocomposites based on a PBAT/PHBV commercial blend was demonstrated, even if issues were raised about the poor interfacial adhesion that currently prevents a full exploitation of the potential of such environmentally friendly composites. The use of compatibilizers has been widely proposed in literature with the aim to increase the interfacial adhesion between natural fillers and polymer matrices. In order to preserve the environmentally friendly character of these biocomposites, a cost-effective solution can rely on the use of natural compatibilizers as an alternative to standard fossil-based compatibilizers. In this regard, vegetable oils are an interesting class of additives that can combine a plasticizing and a compatibilizing effect [18,19,20,21]. In view of all these issues, this work reports for the first time on the effect of two compatibilizers, a maleinized linseed oil (MLO) and a traditional silane, on sustainable composites based on a PBAT/PHBV blend filled with coffee silverskin in both the as-received state and after the extraction of antioxidants. To this purpose, the thermal, mechanical, and morphological properties of injection molded biocomposites were characterized and interpreted as a function of compatibilizer type.

## 2. Materials and Methods

### 2.1. Materials

Coffee silverskin was obtained from a coffee-roasting company located in Rome (Italy) in the form of a blend of 75% (*w*/*w*) Arabica and 25% (*w*/*w*) Robusta. CSS was used in the as-supplied state (CSS_N) and also after extraction of antioxidants (CSS_T). A commercial grade of a biopolymer blend (65% PBAT–35% PHBV) supplied by Nature Plast was used as matrix for biocomposites.

A coupling agent, (3-aminopropyl)triethoxysilane (APTES), supplied by Sigma Aldrich, was used to improve CSS–matrix interactions (CSS_S). A maleinized linseed oil (MLO) provided by Vandeputte (Mouscron, Belgium), commercially known as VEOMER LIN, was used as compatibilizer. This additive is characterized by a viscosity of 10 dPa·s at 20 °C and an acid value of 105–130 mg KOH·g^−1^. 

### 2.2. Methods

#### 2.2.1. Characterization of Coffee Silverskin

Extraction of antioxidants from coffee silverskin was carried out in batch mode using water as solvent. Fifty grams of the material (75% of Arabica and 25% of Robusta) and 1 L of distilled water were loaded into a cylindrical glass vessel (1.5-L working volume) and stirred at 40 ± 0.1 °C and 800 rpm for 60 min. The extractor was provided with a mechanical stirrer and a thermostated water jacket. The resulting suspension was paper filtered and the liquid analyzed for the determination of total phenolics and antioxidant activity. The solid residue was dried in a forced-air dehydrator (Stöckli, Switzerland) operated at 40 °C and stored at room temperature for composite preparation. Moisture content was determined by oven drying at 105 °C to constant weight.

Folin-Ciocalteu method was used to determine the amount of phenolic compounds [22] as gallic acid equivalents (GAE) per unit weight of dry solid using a calibration curve obtained with gallic acid standards. Two different assays, namely DPPH and ABTS [22,23], were used for the assessment of the antioxidant activity of CSS extract. The results were expressed as Trolox equivalents (TE) per unit weight of dry solid using calibration curves obtained with Trolox standards. All the analyses were carried out in duplicate.

EA3000 elemental analyzer (Eurovector, Pavia, Italy) enabled the determination of the elemental composition, while ash content was measured according to ASTM E830 and ASTM D1102. The amount of oxygen was calculated by Equation (1):

*O* (%) = 100 − *C* − *H* − *N* − Ash (1)

The silanization treatment included a preliminary alkali treatment of CSS in an alkali water bath (5% NaOH) for 24 h with constant stirring. Then CSS was drained, washed with distilled water and subsequently dried at 60 °C for 24 h. Silanization was carried out in a water: acetone bath (50:50 *v*/*v*) containing 1% by weight of the corresponding silane with a magnetic stirring for 2 h. After this treatment, the silanized CSS was removed from the bath, drained, and dried at room temperature for 48 h.

Thermogravimetric analysis (TGA) (Seiko Exstar 6300, Tokyo, Japan) was performed to assess the thermal stability of CSS up to 800 °C in a nitrogen atmosphere with a heating rate of 10 °C/min. Fourier transform infrared (FTIR) spectra were recorded on a FTIR spectrometer, JASCO, 680 plus (Easton, MD, USA), by using a KBr-pellet method in the range of 4000–400 cm^−1^ wavenumber.

#### 2.2.2. Production of biocomposites

Composite specimens for mechanical characterization were manufactured by injection molding with a mold temperature kept at 25 °C and an injection temperature of 165 °C with the following pressure cycle: P_injection_ = 6 bar (hold time=0.1 s) and 8 bar (hold time=8.5 s). The different formulations (Table 1) were previously compounded by a vertical twin-screw extruder (DSM Explore 5&15 CC Micro Compounder) with the following parameters: 50 rpm screw speed, 2 min mixing time, and temperature profile: 150–155–160 °C. 

#### 2.2.3. Mechanical Characterization of Biocomposites

Tensile tests were performed in displacement control on a Zwick/Roell Z010 (Kennesaw, GA, USA) with a crosshead speed of 10 mm/min. The dimensions of samples were in agreement with UNI EN ISO527-2 (type 1BA) and a gauge length of 30 mm was used. The results are reported as the average of at least five tests.

#### 2.2.4. Thermal Characterization of Biocomposites

Three samples for each formulation were subjected to differential scanning calorimetry (DSC) using a Mettler Toledo 822e (Columbus, OH, USA) with the following thermal program in nitrogen atmosphere: heating from −20 to 200 °C (5 min hold), cooling to −20 °C (hold time = 5 min), and heating to 200 °C, all steps at 10 °C/min. As PBAT exhibits a very low crystallinity [24], the degree of crystallinity (X_c_) of the composites was calculated according to Equation (2):

(2) $$X_{c}\left(\%\right)=\frac{\Delta H_{m}\left(PHBV\right)-\Delta H_{cc}\left(PHBV\right)}{\Delta H_{m}^{0}\left(PHBV\right)}\frac{100}{w}.$$

where $\Delta H_{m}^{0}$ (PHBV) is the enthalpy of melting of 100% crystalline PHBV (109 J/g) [25], *w* is the weight fraction of PHBV in the blend, Δ*H_m_* represents the experimental enthalpy of melting of the sample (J/g), and Δ*H_cc_* is the enthalpy of cold crystallization.

#### 2.2.5. Morphological Analysis

The filler morphology along with the fracture surfaces of composites failed in tension were investigated by scanning electron microscopy (SEM) using a Hitachi S-2500. All specimens were sputter coated with gold prior to observation.

## 3. Results

### 3.1. Chemical, Thermal and Morphological Analysis of Raw and Treated CSS

The phenolic content of CSS was 7.6 ± 0.21 mg GAE/g_dw_. This value is significantly lower than the one obtained by Conde et al. [26]. This could be attributed to differences in coffee variety and roasting conditions. The CSS blend employed in the present study was richer in Arabica, which, according to Farah [27], has a lower antioxidants content compared to Robusta. In addition, Bessada et al. [16] recently showed that the geographical origin plays a significant role in the chemical composition of silverskin obtained from *Coffea canephora* beans, especially in terms of fatty acid profile and antioxidant composition. Furthermore, the treatment was carried out under mild temperature conditions to preserve the activity of the antioxidant compounds. Moreover, this value represents the 47% of the total phenolic content obtained by Sarasini et al. [17] employing the same CSS blend. The extraction yield obtained is quite low. This could be attributed to the used extraction conditions. In fact, according to Zuorro and Lavecchia [28], the affinity of many antioxidants for hydro-alcoholic solvents is higher than for pure water. However, in the present study, the treatment of CSS with water was required in order to remove the water-soluble components and improve the affinity with a polymer matrix. In fact, Zarrinbakhsh et al. [29] showed that a water-based green surface treatment of distiller’s dried grains with solubles (DDGS), a major coproduct of the corn ethanol industry, led to a noticeable improvement in the degradation onset temperature of DDGS due to the elimination of many water-soluble components, improving the resistance of the material during melt processing of the composite. Moreover, the PHBV-based bioplastic composite obtained with water treated DDGS showed an enhanced modulus and an improved adhesion between the matrix and filler compared to the composites produced with untreated material. The removal of water-soluble compounds was confirmed in this study by the results of elemental analysis. Ash content of water-extracted CSS was 4.55% ± 0.07% (*w*/*w*). The elemental composition calculated on a dry and ash-free basis (% *w*/*w*) was as follows: C = 49.43 ± 0.40, H = 6.45 ± 0.13, N = 7.41 ± 0.10, O = 36.71 ± 1.31. These values lead to an atomic oxygen-to-carbon ratio of 0.56, which is lower than that obtained for the untreated material [17], confirming that the amount of hydroxyl groups in the treated material decreased.

The antioxidant activity of CSS extract as determined by DPPH and ABTS was 3.87 ± 0.23 and 5.82 ± 0.29 mg TE/g_dw_, respectively. These two methods are based on different reactions, thus leading to different values [30]. The antioxidant capacity of CSS can be mainly ascribed to the presence of polyphenols. Other bioactive compounds can contribute to the antioxidant capacity of CSS, such as melanoidins, which are formed during the roasting of green coffee beans as a product of the Maillard reaction [31].

**Figure 1:**
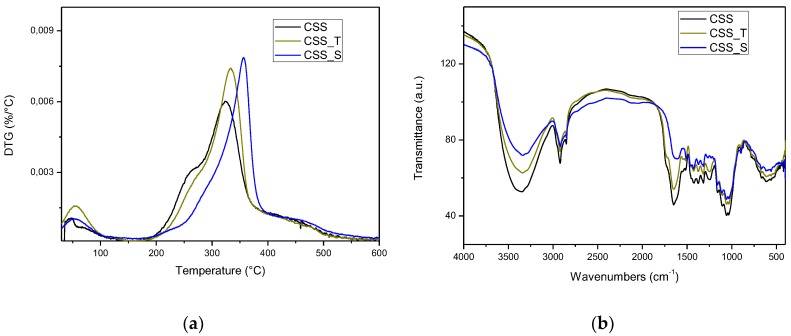
Derivative thermograms (DTG) curves (**a**) and Fourier Transform Infrared (FTIR) spectra (**b**) of as received, treated, and silane-treated coffee silverskin (CSS).

Derivative thermograms (DTG) under nitrogen atmosphere for untreated, treated, and silane- modified coffee silverskin are reported in Figure 1a, while the FTIR spectra of as-received, treated, and silane-modified CSS are reported in Figure 1b. A typical three-step thermal degradation occurred: the first one, up to about 130–135°C, can be related to moisture removal, after that the maximum degradation rate, due to hemicellulose and cellulose decomposition, occurred at 336.5, 334, and 357 °C, respectively, for CSS, CSS_T, and CSS_S. Comparing the degradation rates of CSS and CSS_T, the latter was found to be more thermally stable, reflecting that less damaged hemicelluloses existed, pyrolysed at higher temperature [32]. In line with previous results on thermal stability of silane-treated natural fibers, silane treatment improved the thermal stability of the fiber by removing pectin and wax and exposing higher amount of cellulose [33].

FTIR spectrum of CSS reported in Figure 1b exhibited the typical absorption bands of lignocellulosic materials, as already observed in [17]. While no substantial differences were found in the case of CSS_T, the silane treatment removed pectin and wax, as attested by the absence of peaks at 1731 and 1253 cm^−1^. 

The broad band at 3354 cm^−1^ is characteristic of O–H stretching for hydroxyl groups in polysaccharide chains. It became less intense for the extracted CSS, confirming the results of elemental analysis. Similarly, as a result of the silane treatment, the hydrophilic character of the CSS was reduced and the water content decreased. Water also shows a bending vibration at around 1650 cm^−1^, that was less intense (or completely absent in the case of the signal at 1545 cm^−1^) in silane- treated CSS [34].

**Figure 2:**
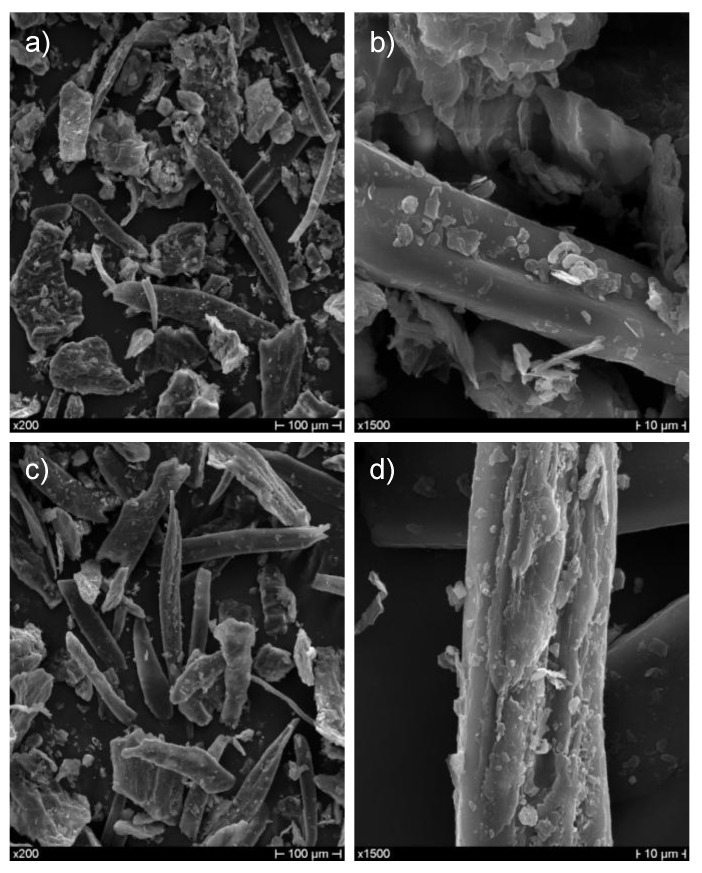
SEM micrographs of (**a**,**b**) as-received and (**c**,**d**) extracted CSS.

As regards the morphology of as-received and extracted CSS, no substantial differences can be noted from micrographs obtained by scanning electron microscopy (Figure 2). It is evident that the flaky structure typical of coffee silverskin [17] has been partially dismantled and its fibrous structure resulted in the occurrence of several isolated fibers with diameters of tens of microns, further supporting its use as reinforcement in polymer composites.

### 3.2. Tensile Properties of Composites

**Figure 3:**
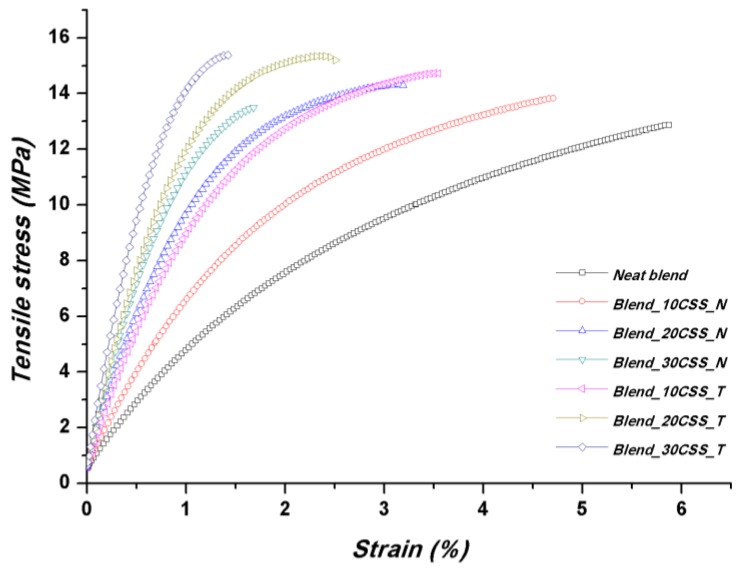
Typical stress–strain curves from tensile tests of PBAT/PHBV-based composites with non-compatibilized CSS.

With regard to the tensile properties, the incorporation of CSS into PBAT/PHBV blend produced a substantial increase of the elastic modulus values, as can be inferred from the stress vs. strain curves shown in Figure 3 and the corresponding mechanical properties summarized in Table 2.

While this behavior is rather common due to the stiff lignocellulosic fillers that prevent molecular mobility of polymer chains, interestingly, the tensile strength of neat blend was not degraded, indeed was slightly improved by the addition of CSS, even if composites with a content of 30 percent by weight appear to have reached a kind of saturation, likely due to a non-optimal distribution of the fillers in the molten polymer during compounding. As already commented on in [17], the tensile strength remained almost even after the incorporation of up to 30 wt % of untreated coffee silverskin while, as expected, modulus of the composites increased progressively with increasing filler content. In particular, the values of elastic moduli reached 3.7 and 4.4 times the reference (neat blend) in the case, respectively, of Blend_30CSST_S and Blend_30CSS_T systems, whereas the improvement for untreated and MLO modified blend was 3.3 and 3.1 times the neat matrix. This result is widely over the performance of a previous studied PP based system containing CSS [10], where both a limited increase of elastic modulus and a general decrease of tensile strength were observed. In our case, all the produced blends with the different CSS at distinct content and modifications showed a general, even limited, increase. 

**Figure 4:**
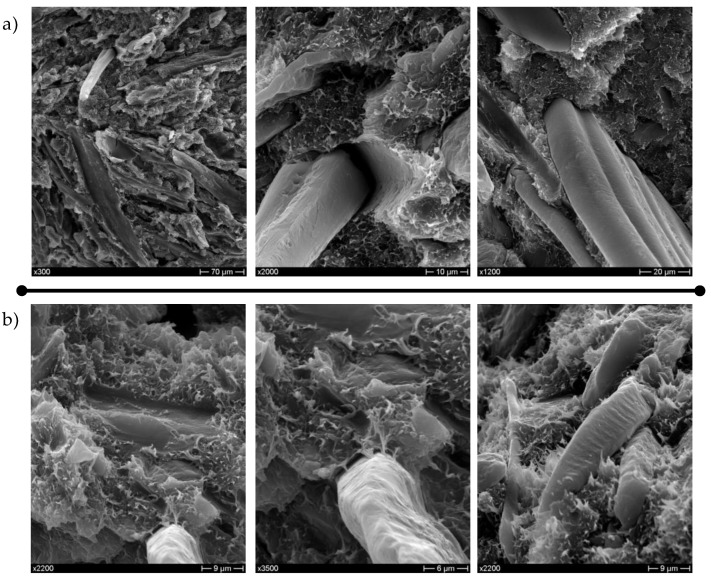
SEM micrographs of biocomposites with 30 wt % CSS in the as-received state (**a**) and with 30 wt % CSS after extraction (**b**) at different magnifications.

On the other hand, extracted CSS is more effective than as-received CSS, which is to be ascribed to a sounder interfacial adhesion, as confirmed by SEM investigation of the fracture surfaces shown in Figure 4a,b. Composites with untreated CSS (Figure 4a) exhibited a poor interfacial adhesion with extensive fiber pull-out and debonding at filler/matrix interface, while the extracted CSS fibers/particles (Figure 4b) appear to be more embedded within the matrix with the presence of polymer ligaments connecting them to the matrix. This different behavior can be ascribed to the removal of water-soluble components during the extraction process. However, the injection-molded composites also became more brittle as the elongation at break was severely impaired by the increasing content of CSS, irrespective of surface modification. Neat blend, as previously reported [17], is characterized by mixed mode fracture morphology, with both ductile and relatively brittle zones due to the different deformation behavior of the single constituents. In composite materials, a limited ductility can be only observed at the micro scale (Figure 4a,b).

These mechanical results are somewhat different from the usual ones regarding the mechanical response of agricultural residues when added to several polymer matrices [21,35,36,37,38], where lower tensile strength compared to the neat matrix is generally reported in not-compatibilized composite systems.

**Figure 5:**
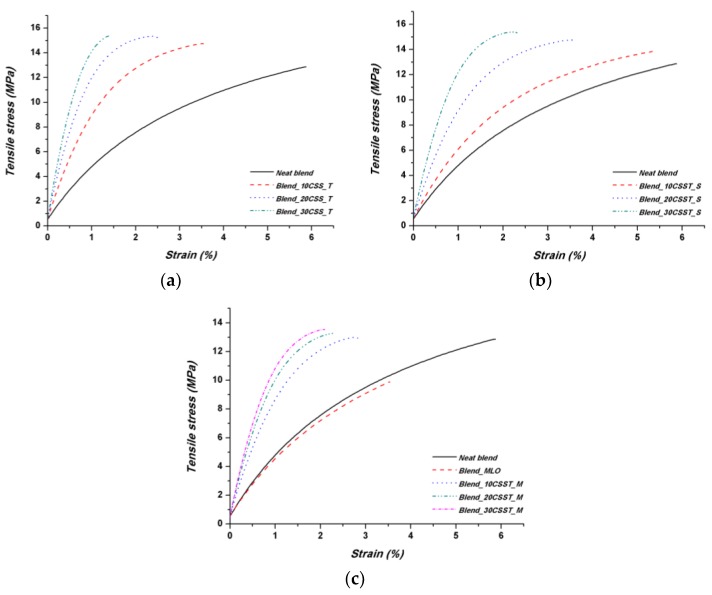
Typical stress–strain curves from tensile tests of PBAT/PHBV-based composites with extracted CSS (**a**); silane-treated CSS (**b**); and maleinized linseed oil (MLO) modifier (**c**) at different CSS contents.

With regard to the effect of compatibilizers, maleinized linseed oil was found not to improve the overall mechanical behavior, including the elongation at break (Figure 5 and Table 2). 

**Figure 6:**
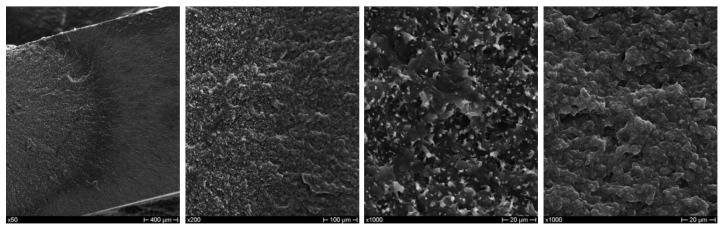
SEM micrographs of the polymer blend compatibilized with MLO at different magnifications.

MLO and other vegetable oils have been reported as effective plasticizers in PLA and its blends with TPS, PCL, PHB [18,19,39,40,41], allowing chain motion and improving their processing conditions. In all these systems, a substantial decrease in strength and stiffness is usually combined with a marked increase in ductility. In the present case, even for the blend modified with MLO, this decrease in strength properties was not accompanied by an improved ductility as confirmed also by the observation of fracture surface (Figure 6), which showed features similar to the ones of neat blend, i.e. the coexistence of ductile and brittle areas. It is reasonable to assume that MLO is not particularly effective in the case of PBAT/PHBV blend, considering that its efficiency on poly (3-hydroxybutyrate) is reported to be not as high as that for PLA [39].

**Figure 7:**
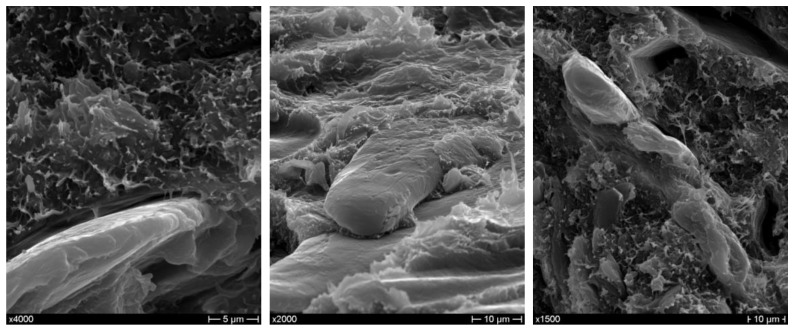
SEM micrographs of biocomposites with 30 wt % CSS after extraction and MLO at different magnifications.

The presence of extracted CSS counteracted this low efficiency resulting in composites with mechanical properties higher (especially for modulus) than the neat blend, partially ascribed to a better interfacial compatibility, as can be inferred from SEM micrographs (Figure 7).

In fact MLO, with its highly reactive maleic anhydride functionality, can potentially provide a compatibilization effect with CSS, due to the formation of ester bonds between the multiple MAH groups and the hydroxyl groups of CSS. In the present case, this effect seems to be present but limited by the fact that MLO was added in formulations based on extracted CSS, characterized by a reduced amount of hydroxyl groups.

**Figure 8:**
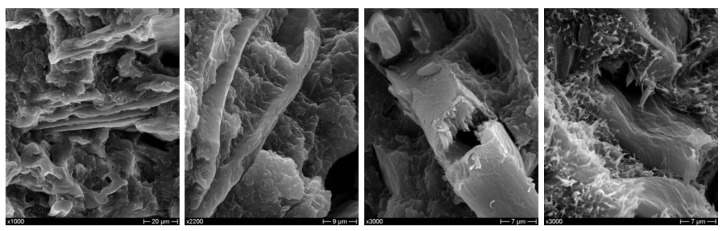
SEM micrographs of biocomposites with 30 wt % CSS after extraction and surface modification with silane at different magnifications.

Interestingly, the modification of CSS with silane provided the best combination of resistance properties and ductility (Figure 5 and Table 2). Silane induced an improved interfacial adhesion [42], as can be seen in Figure 8, which allowed for an efficient stress transfer between the polymer and the filler with occurrence of filler breakage. The increased ductility can be related to a better filler wettability and dispersion achieved through compatibilization [43], thus reducing the presence of filler clusters that can act as points of stress concentration.

### 3.3. Thermal properties of composites

**Figure 9:**
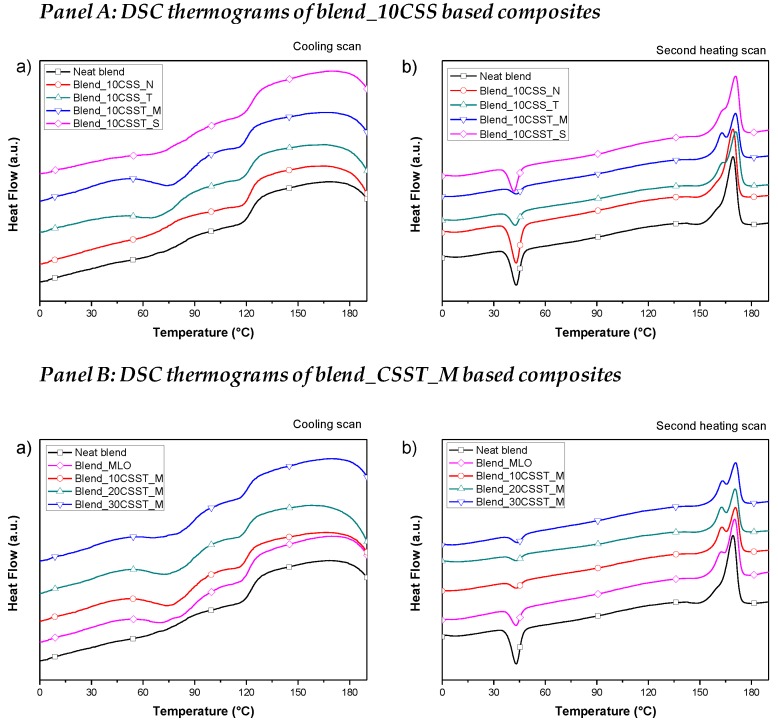
DSC curves for neat PBAT/PBVH and blend_10CSS based composites (Panel A) and for neat PBAT/PBVH, blend_MLO and blend_CSST_M based composites (Panel B) during cooling (**a**) and second heating (**b**) scans.

The thermal properties of PBAT/PHBV blend-based formulations were analyzed, with the aim of investigating the effect of the incorporation of as received, treated and silane-modified CSS into PBAT/PHBV blends. Specifically, DSC cooling and heating scans were performed to evaluate the effect of different components on crystallization and melting profile. Table 3 summarizes the data obtained from the cooling and heating scans for all the produced formulations. Figure 9 shows the DSC thermograms for neat PBAT/PBVH and Blend_10CSS-based composites (Figure 9, Panel A) and for neat PBAT/PBVH and Blend_CSST_M -based composites (Figure 9, Panel B) during cooling (a) and second heating (b) scans.

In the cooling scan measurements, it is possible to note that a slight variation of crystallization temperature relative to PBAT component (*T*_cPBAT_) and a negligible variation regarding the crystallization temperature of PHBV component (*T*_cPHBV_) were measured for Blend_CSS_N based composites. Furthermore, thermal characterization studies showed a higher crystallization temperature (*T*_cPBAT_) in Blend_CSST_M based composites with increasing content of CSST_M component. It is reasonable to observe and assume that MLO modified the thermal properties of the neat blend. Figure 9b (Panel A and Panel B) shows that neat blend and its composites are characterized by the presence of double crystallization peaks located nearly at the same temperature, this phenomenon underlines that the presence of different CSS components did not affect the size and the lamellar structures achieved during the crystallization process of the realized biocomposites [17,44].

Neat blend and biocomposites exhibited cold crystallization peaks, indicating that there were present amorphous areas able to crystallize [17], and this behavior was also observed for other biobased polymers [45,46]. A slight decrease of cold crystallization temperature (*T*_cc_) was registered for Blend_CSST_S based formulations with respect to the neat blend and other composites, while a slight increase of T_cc_ was observed for Blend_CSST_M systems. In addition, the cold crystallization enthalpy (Δ*H*_cc_) values decrease with increasing amount of CSS in biocomposites, as reported in Table 3 and Figure 9 (Panel A and Panel B). The lower value of Δ*H*_cc_ was measured for Blend_30CSST_S, this effect indicated that CSS based systems presented reduced amorphous regions able to crystallize in second heating scan. This means that silane treatment for CSS was responsible of a reduced macromolecular flexibility and mobility upon increasing temperature for the blend, as a result of increased interfacial adhesion with surface treated CSS.

Nevertheless, small variations for the *T*_g_ values have been detected in presence of MLO when combined with treated and silane-modified CSS: neat Blend exhibited a *T*_g_ value of 42.4 °C, which slightly decreased to 40.7 °C for Blend MLO. The addition of increasing content of CSS_T in MLO modified blends did not essentially change the *T*_g_ values (from 40.1 to 39.3 °C, for Blend_10CSST_M and Blend_30CSST_M, respectively), meaning that the consequence of silane modification was more effective that the introduction of plasticizing agent.

No particular variations were registered for the melting temperatures of PBAT and PHBV components using the different CSS and amount with respect to the neat blend. The melting peaks of PBAT (melting enthalpy Δ*H*_m PBAT_) in composite formulations (Table 3 and Figure 9 (Panel A and Panel B)) showed decreased values with respect to neat blend, while the melting enthalpy of PHBV was not considerably affected [17]. It is concluded that the nucleation of crystals that melt at high temperature is prevented by the filler, while the development of crystals able to melt at lower temperature is favored. This behavior depends on the effect that CSS has on crystallization: it enables the crystallization of the low melting component, acting as nucleating agent, but it does not affect the crystallization behavior of high melting phase.

However, it has been measured that the incorporation of CSS in the selected biobased polymeric blend (PHBV/PBAT) determines an increase of the degree of crystallinity (X_c_) over the range of different produced and characterized formulations (Table 3), in accordance with other research activities dealing with the use of recycled wood materials, nanoclays and coffee silverskin [17,24,25]. The crystallization phenomenon/behavior can be also correlated, in combination with the higher aspect ratio, to the reported results on strength properties and Young’s modulus of neat blend and biocomposites with different typologies of CSS and content (Figure 3, Figure 5 and Table 2): the modification of CSS with silane represented the best combination of tensile strength and ductility (Figure 5 and Table 2), in parallel with the higher values for *X*_c_.

## 4. Conclusions

In the present study, the use of coffee silverskin in as-received state (CSS_N), after polyphenols extraction (CSS_T) and silane treatment (CSS_S) as suitable filler for PBAT/PHBV blends was investigated. Furthermore, the plasticizing effect of a maleinized linseed oil (MLO) was considered as a possible solution for tuning the mechanical response of composites at three different CSS amounts (10, 20 and 30 wt %). The resulting composites, manufactured by a common melt blending process, exhibited a reasonable combination of thermal and mechanical properties. In details, the incorporation of CSS into PBAT/PHBV blend produced a substantial increase in the elastic modulus values: extracted CSS and silane-treated CSS provided the best combination of resistance properties and ductility, due to a sounder interfacial adhesion, while MLO provided a limited compatibilization effect with CSS, due to the reduced amount of hydroxyl groups on CSS after extraction. Silane treatment for CSS was also responsible for a reduced macromolecular flexibility and mobility upon increasing temperature for the blend, on the other hand small variations in the *T*_g_ values were detected in the presence of MLO when combined with treated and silane-modified CSS, suggesting that the effects of silane modification were more evident than the introduction of plasticizing agent. The improvement of interface compatibility between CSS and biopolymers is indeed an issue of fundamental importance to fully exploit the potential of such composites in different applications.

## Figures and Tables

**Table 1:** Summary of the formulations investigated in the present study.

Specimen ID	Matrix (PBAT/PHBV) (wt %)	As-received CSS (wt %)	Extracted CSS (wt %)	Maleinized Linseed Oil (wt %)	Silane- Treated CSS (wt %)
Neat blend	100	-	-	-	-
Blend_10CSS_N	90	10	-	-	-
Blend_20CSS_N	80	20	-	-	-
Blend_30CSS_N	70	30	-	-	-
Blend_10CSS_T	90	-	10	-	-
Blend_20CSS_T	80	-	20	-	-
Blend_30CSS_T	70	-	30	-	-
Blend_MLO	97	-	-	3	-
Blend_10CSST_M	87	-	10	3	-
Blend_20CSST_M	77	-	20	3	-
Blend_30CSST_M	67	-	30	3	-
Blend_10CSST_S	90	-	-	-	10
Blend_20CSST_S	80	-	-	-	20
Blend_30CSST_S	70	-	-	-	30

**Table 2:** Summary of tensile properties for PBAT/PHBV-based composites.

Specimen ID	Tensile Strength (MPa)	Young’s Modulus (MPa)	Strain at Failure (%)
Neat blend	12.90 ± 0.83	429 ± 30	5.92 ± 0.54
Blend_10CSS_N	13.70 ± 0.44	663 ± 62	4.56 ± 0.15
Blend_20CSS_N	14.33 ± 0.15	1164 ± 133	3.25 ± 0.26
Blend_30CSS_N	13.52 ± 0.31	1432 ± 118	1.80 ± 0.27
Blend_10CSS_T	14.82 ± 0.26	1015 ± 70	3.57 ± 0.39
Blend_20CSS_T	15.30 ± 0.19	1503 ± 52	2.45 ± 0.14
Blend_30CSS_T	15.01 ± 0.75	1902 ± 101	1.55 ± 0.32
Blend_MLO	9.88 ± 0.27	394 ± 11	3.67 ± 0.16
Blend_10CSST_M	12.86 ± 0.41	656 ± 69	2.91 ± 0.09
Blend_20CSST_M	13.32 ± 0.28	1317 ± 84	2.14 ± 0.36
Blend_30CSST_M	13.08 ± 0.37	1331 ± 80	2.24 ± 0.12
Blend_10CSST_S	13.74 ± 0.34	617 ± 13	5.07 ± 0.57
Blend_20CSST_S	14.71 ± 0.22	1034 ± 39	3.76 ± 0.20
Blend_30CSST_S	15.38 ± 0.11	1566 ± 51	2.26 ± 0.09

**Table 3:** Differential scanning calorimetry (DSC) results of PBAT/PHBV blend-based formulations.

Specimen ID	Cooling Scan				Second Heating Scan							
	*T*_c PBAT_ (°C)	Δ*H*_c PBAT_ (J/g)	T_c PHBV_ (°C)	Δ*H*_c PHBV_ (J/g)	*T*_cc PHBV_ (°C)	Δ*H*_cc PHBV_ (J/g)	*T*_m PBAT_ (°C)	Δ*H*_m _PBAT_ (J/g)	*T*_m1_PHBV_ (°C)	*T*_m2_PHBV_ (°C)	Δ*H*_m PHBV_ (J/g)	*X*_c PBHV_ (%)
**Neat blend**	72.1 ± 1.3	2.5 ± 0.1	117.9 ± 1.2	4.0 ± 0.1	43.0 ± 1.2	9.0 ± 0.5	129.6 ± 1.2	6.4 ± 0.3	160.2 ± 0.8	169.3 ± 0.5	23.8 ± 1.1	38.7 ± 0.5
**Blend_10CSS_N**	69.4 ± 1.1	1.0 ± 0.2	117.7 ± 1.3	4.5 ± 0.2	43.0 ± 1.1	10.1 ± 0.6	127.8 ± 1.1	5.0 ± 0.2	160.3 ± 0.8	169.1 ± 0.9	23.4 ± 0.8	38.8 ± 0.4
**Blend_20CSS_N**	64.5 ± 0.5	3.5 ± 0.3	116.0 ± 1.2	4.0 ± 0.2	44.2 ± 1.1	5.3 ± 0.3	129.2 ± 0.9	5.2 ± 0.3	162.9 ± 0.5	171.4 ± 0.8	19.7 ± 1.2	47.4 ± 0.6
**Blend_30CSS_N**	66.1 ± 0.6	2.1 ± 0.2	115.1 ± 1.1	3.9 ± 0.3	44.6 ± 1.2	6.1 ± 0.4	130.3 ± 1.3	4.4 ± 0.2	160.3 ± 0.4	169.8 ± 0.4	18.2 ± 1.2	45.2 ± 0.7
**Blend_10CSS_T**	68.13 ± 0.4	4.1 ± 0.2	116.4 ± 1.2	3.5 ± 0.2	42.7± 0.5	3.5 ± 0.4	129.2 ± 1.2	3.2 ± 0.3	163.0 ± 0.9	171.0 ± 0.5	20.9 ± 0.4	50.6 ± 0.8
**Blend_20CSS_T**	66.0 ± 0.3	3.1 ± 0.2	116.9 ± 1.5	3.1 ± 0.1	42.8 ± 0.2	3.1 ± 0.2	129.7 ± 0.5	3.0 ± 0.2	161.9 ± 1.2	170.5 ± 0.4	18.4 ± 0.2	50.3 ± 1.5
**Blend_30CSS_T**	79.9 ± 1.2	4.7 ± 0.5	116.5 ± 1.5	2.0 ± 0.1	43.9 ± 0.4	2.1 ± 0.1	130.2 ± 1.5	2.5 ± 0.1	162.7 ± 1.1	170.7 ± 0.5	17.2 ± 0.4	56.7 ± 2.2
**Blend_MLO**	72.3 ± 0.6	5.1 ± 0.5	117.9 ± 1.8	2.9 ± 0.2	43.1 ± 0.5	4.3 ± 0.3	128.7 ± 0.8	3.0 ± 0.2	162.3 ± 1.3	170.2 ± 1.1	22.6 ± 0.4	49.5 ± 1.8
**Blend_10CSST_M**	77.8 ± 0.2	8.6 ± 0.7	116.1 ± 0.5	2.2 ± 0.1	43.5 ± 0.4	1.8 ± 0.1	126.8 ± 1.1	2.8 ± 0.2	162.7 ± 0.4	170.7 ± 1.2	18.8 ± 0.5	51.1 ± 1.7
**Blend_20CSST_M**	75.8 ± 0.3	6.8 ± 0.4	115.7 ± 0.7	2.0 ± 0.1	43.7 ± 0.7	1.5 ± 0.1	128.0 ± 1.2	3.8 ± 0.5	162.5 ± 0.5	170.4 ± 0.7	17.9 ± 0.6	55.9 ± 2.3
**Blend_30CSST_M**	79.9 ± 0.4	5.7 ± 0.2	116.5 ± 0.5	2.3 ± 0.1	44.4 ± 0.6	2.0 ± 0.1	129.4 ± 0.4	2.7 ± 0.2	163.3 ± 0.6	170.7 ± 0.5	17.3 ± 0.3	59.3 ± 2.5
**Blend_10CSST_S**	70.7 ± 1.2	3.7 ± 0.2	117.1 ± 1.1	3.5 ± 0.5	41.7 ± 0.4	5.9 ± 0.3	129.7 ± 0.4	3.6 ± 0.1	162.1 ± 0.5	170.9 ± 0.5	22.2 ± 1.1	47.4 ± 2.4
**Blend_20CSST_S**	70.7 ± 1.5	1.8 ± 0.1	117.2 ± 1.2	3.3 ± 0.2	41.7 ± 0.8	3.8 ± 0.2	129.2 ± 0.8	3.6 ± 0.2	163.0 ± 0.8	170.6 ± 0.7	21.0 ± 1.2	56.1 ± 2.7
**Blend_30CSST_S**	73.4 ± 1.2	2.8 ± 0.2	118.5 ± 1.5	3.1 ± 0.2	41.4 ± 0.5	1.3 ± 0.1	131.8 ± 1.1	3.3 ± 0.1	163.3 ± 0.6	170.7 ± 0.4	18.2 ± 0.5	63.2 ± 2.4
